# Reports of police beating and associated harms among people who inject drugs in Bangkok, Thailand: a serial cross-sectional study

**DOI:** 10.1186/1471-2458-13-733

**Published:** 2013-08-07

**Authors:** Kanna Hayashi, Lianping Ti, Joanne Csete, Karyn Kaplan, Paisan Suwannawong, Evan Wood, Thomas Kerr

**Affiliations:** 1 Urban Health Research Initiative, British Columbia Centre for Excellence in HIV/AIDS, St. Paul’s Hospital, 608-1081 Burrard Street, Vancouver, BC V6Z 1Y6, Canada; 2 Interdisciplinary Studies Graduate Program, University of British Columbia, Green Commons, 6201 Cecil Green Park Road, Vancouver, BC V6T 1Z1, Canada; 3 School of Population & Public Health, University of British Columbia, 2206 East Mall, Vancouver, BC V6T 1Z3, Canada; 4 Global Drug Policy Program, Open Society Foundations, 7th Floor Millbank Tower, 21-24 Millbank, London SW1P 4QP, United Kingdom; 5 Mitsampan Harm Reduction Center / Thai AIDS Treatment Action Group, 18/89 Vipawadee Rd., soi 40 Chatuchak, Bangkok 10900, Thailand; 6 Faculty of Medicine, University of British Columbia, 317-2194 Health Sciences Mall, Vancouver, BC V6T 1Z3, Canada

**Keywords:** HIV/AIDS, Drug law enforcement, Injection drug use, Harm reduction, Thailand

## Abstract

**Background:**

Thailand has for years attempted to address illicit drug use through aggressive drug law enforcement. Despite accounts of widespread violence by police against people who inject drugs (IDU), the impact of police violence has not been well investigated. In the wake of an intensified police crackdown in 2011, we sought to identify the prevalence and correlates of experiencing police beating among IDU in Bangkok.

**Methods:**

Community-recruited samples of IDU in Bangkok were surveyed between June 2009 and October 2011. Multivariate log-binomial regression was used to identify factors associated with reporting police beating.

**Results:**

In total, 639 unique IDU participated in this serial cross-sectional study, with 240 (37.6%) participants reporting that they had been beaten by police. In multivariate analyses, reports of police beating were associated with male gender (Adjusted Prevalence Ratio [APR] = 4.43), younger age (APR = 1.69), reporting barriers to accessing healthcare (APR = 1.23), and a history of incarceration (APR = 2.51), compulsory drug detention (APR = 1.22) and syringe sharing (APR = 1.44), and study enrolment in 2011 (APR = 1.27) (all *p* < 0.05). Participants most commonly reported police beating during the interrogation process.

**Conclusions:**

A high proportion of IDU in Bangkok reported having been beaten by the police. Experiencing police beating was independently associated with various indicators of drug-related harm. These findings suggest that the over-reliance on enforcement-based approaches is contributing to police-perpetrated abuses and the perpetuation of the HIV risk behaviour among Thai IDU.

## Background

In many countries, illicit drug use remains a significant problem, and the dominant response continues to be the enforcement of drug laws that criminalize illicit drug use and trafficking [1]. However, a burgeoning body of international literature suggests that the overreliance on law enforcement-based approaches produces unintended negative consequences, including the perpetuation of human immunodeficiency virus (HIV) epidemics among people who inject drugs (IDU), as these approaches have been associated with an unwillingness to carry sterile syringes and risky injection behaviour among this population [2-4]. The reliance on drug law enforcement continues despite international guidelines issued by agencies of the United Nations that recommend a public health approach to problematic drug use (e.g., harm reduction services such as sterile syringe programs) [5].

Thailand has experienced longstanding epidemics of illicit drug use and HIV among IDU, with an estimated 5% of the population using illicit drugs in 2007 and an estimated 30–50% of IDU living with HIV/AIDS over two decades [6,7]. Although Thailand enacted a new law that reclassified people who use drugs as “patients” not “criminals” in 2002, the criminal laws governing drug use remain in effect, and the Thai government has continued to support intensive police crackdowns, as well as compulsory detention and incarceration of people who use drugs [8]. Between 2008 and 2011, Thai drug policies were revised several times, and the number of people who use drugs targeted to undergo mandatory rehabilitation programs has increased from 60,000 in 2008 to 400,000 in 2011 [9-12]. It has been reported that during the Thai “war on drugs” in 2003, the government’s strong emphasis on drug suppression efforts led the Thai police to commit various forms of violence, including over 2,800 extrajudicial killings of alleged drug dealers and users [13,14]. Although the Thai government promised that the police would not breach due process again, recent reports suggest that police misconduct has continued during subsequent crackdowns. For example, a 2008 study showed that almost half of a sample of IDU in Bangkok reported having drugs planted on them by police [15]. Further, other reports documented police misconduct and fatal shootings of suspects during drug suppression operations in 2012 [16,17].

Despite ongoing concern regarding the renewed and intensified crackdowns on drug use in Thailand, few studies have endeavored to identify the extent and impact of specific forms of police violence among IDU. Although many detailed narratives on police violence were documented during the “war on drugs” in 2003 [13], few studies have been undertaken in the post-2003 period [15,18]. As well, while international literature indicates that aggressive drug law enforcement practices increase vulnerability to HIV infection and other harms among IDU [4,19-21], these studies have tended to focus on the aggregate impact of police crackdowns of relatively short duration, and the impact of specific forms of police misconduct has seldom been quantified. Moreover, most of the previous academic research in this field has been conducted in Western countries, not in Southeast Asia where the legal and social environments surrounding illicit drug use are distinct from those in Western settings [22]. Therefore, we sought to identify the prevalence and correlates of experiencing police beating among a community-recruited sample of IDU in Bangkok, Thailand.

## Methods

### Study design

Data for this study were derived from the Mitsampan Community Research Project, a collaborative research effort involving the Mitsampan Harm Reduction Center (MSHRC; a drug user-run drop-in centre in Bangkok, Thailand), Thai AIDS Treatment Action Group (Bangkok, Thailand), Chulalongkorn University (Bangkok, Thailand), and the British Columbia Centre for Excellence in HIV/AIDS/University of British Columbia (Vancouver, Canada). This is a serial cross-sectional study that aims to investigate drug-using behaviour, healthcare access, and other drug-related harms among IDU in Bangkok. Between June 2009 and October 2011, the research partners undertook two waves of surveying, which involved a total of 757 community-recruited IDU in Bangkok (317 IDU between June and July of 2009 and 440 IDU between July and October of 2011). Potential participants were recruited through peer outreach efforts and word-of-mouth, and were invited to attend the MSHRC or O-Zone House (another drop-in centre in Bangkok) in order to be part of the study. Adults residing in Bangkok or in adjacent provinces who had injected drug(s) in the past six months were eligible for participation.

All participants provided oral informed consent and completed an interviewer-administered questionnaire eliciting a range of information, including socio-demographic characteristics, drug use patterns, and experiences with drug law enforcement and accessing healthcare. Most of the question items used for this study were consistent with those utilized in previous studies of IDU in Bangkok [23,24]. All interviews were conducted in private rooms inside the MSHRC and O-Zone House by a total of 11 trained peer interviewers (i.e., former or active drug users at the MSHRC, including seven interviewers in 2009 and six interviewers in 2011), who underwent extensive training focused on interview techniques and research ethics. In addition, research staff checked the administered questionnaires for consistency across both interviews and interviewers on an ongoing basis. Each interview lasted between 45 and 90 minutes. Upon completion of the questionnaire, participants received a stipend of 350 Thai Baht (approximately US$12). The study was approved by the research ethics boards at Chulalongkorn University and the University of British Columbia.

### Participants and measures

Participants who completed the interview in 2009 or 2011 were eligible for inclusion. Given that some individuals were interviewed in both 2009 and 2011, we included all participants from the first wave and only new participants from the second wave in order to ensure the independence of the observations analysed in the present study. The sample of each survey wave was further restricted to individuals who had complete data for the present analyses. For the present analyses, the primary outcome of interest was reporting a history of police beating, defined as answering “Yes” to a question: “Have you ever been beaten by police?” In addition, in 2011 a follow-up question was added to the survey, which asked participants reporting episodes of police beating about the circumstances of police beatings (e.g., where and when they occurred). Although many forms of police violence exist [25], police beating was selected as the primary outcome of interest because it was identified as one of the major forms of police violence by a group of peer researchers during the process of developing survey instruments, and yet little was known about the prevalence and correlates of experiencing police beating among IDU in Bangkok.

Informed by previous studies exploring the impacts of aggressive drug law enforcement on IDU [19-21], explanatory variables that were hypothesized to be potentially associated with the outcome were selected. They included median age (< 37 years *vs.* ≥ 37 years); gender (male *vs.* female); drug-dealing involvement in the past six months (yes *vs.* no); a history of injecting each of the four kinds of drugs that are commonly used among IDU in Bangkok: heroin, midazolam (a short-acting benzodiazepine), methamphetamine (locally called *yaba*), and crystal methamphetamine (locally called *ice*); a history of incarceration; a history of compulsory drug detention; a history of accessing methadone treatment; reporting barriers to accessing healthcare (any *vs.* none); a history of syringe sharing; HIV serostatus (positive *vs.* negative or unknown); a history of non-fatal overdose; and calendar year of study enrolment (2011 *vs.* 2009). Drug-dealing involvement was ascertained by asking whether drug dealing (i.e., selling or delivering illicit drugs) constituted a source of personal income for the respondent in the past six months. As in our previous work [24], our barriers to accessing healthcare variable included a range of potential barriers, including but not limited to: fear of sharing information of drug using status with the police, not wanting healthcare providers to know one injects or uses drugs, being treated poorly by healthcare providers, and transportation issues.

### Statistical analyses

For the bivariate and multivariate analyses, the prevalence ratio was used as a measure of association, rather than the odds ratio, as the frequency of the outcome exceeded 10% [26]. First, we used a simple binomial regression with a log link function to examine bivariate associations between reports of police beating and the explanatory variables [27]. Next, we used an *a priori*-defined statistical protocol based on examination of the Akaike Information Criterion (AIC) and *p*-values to construct an explanatory multivariate log-binomial regression model using the COPY method in SAS (SAS Institute Inc., Cary, NC, USA) [28]. First, we constructed a full model including all variables analysed in bivariate analyses. After examining the AIC of the model, we removed the variable with the largest *p*-value and built a reduced model. We continued this iterative process until no variables remained for inclusion. We selected the multivariate model with the lowest AIC score. Because median age, which was not significantly associated with the outcome at the *p* < 0.05 level in bivariate analyses, became significantly associated with the outcome in the full multivariate log-binomial regression model, we assessed two-way interactions between median age and the explanatory variables by creating interaction terms. Interaction was deemed as present if the interaction term was associated with the outcome at the *p* < 0.05 level. All *p*-values were two-sided.

As a sub-analysis, we used descriptive statistics to examine at what point during interactions with police that participants experienced beatings. All statistical analyses were performed with SAS version 9.3.

## Results

Among 644 unique IDU recruited between June 2009 and October 2011, 639 individuals (307 individuals in 2009 and 332 individuals in 2011) had complete data and were eligible for inclusion in the present analyses. The median age of eligible participants was 37 years (interquartile range: 33–47 years), and 153 (23.9%) were female. In total, 240 participants (37.6%) reported having ever been beaten by police. The unadjusted prevalence of experiencing police beating increased from 31.3% in 2009 to 43.4% in 2011 (*p* = 0.002). Twenty-one individuals (3.3%) reported having been beaten by police in the past six months (6 individuals [2.0%] in 2009 and 15 individuals [4.5%] in 2011; *p* = 0.082).

The results of bivariate analyses are shown in Table 1. Reports of police beatings were significantly and positively associated with male gender (prevalence ratio [PR]: 5.50; 95% confidence interval [CI]: 3.24–9.33); a history of heroin injection (PR: 2.45; 95% CI: 1.45–4.15), midazolam injection (PR: 1.52; 95% CI: 1.10–2.10), and crystal methamphetamine injection (PR: 1.47; 95% CI: 1.17–1.84); a history of syringe sharing (PR: 1.93; 95% CI: 1.52–2.45); a history of incarceration (PR: 3.00; 95% CI: 1.98–4.56); a history of compulsory drug detention (PR: 1.33; 95% CI: 1.06–1.66); a history of methadone treatment enrolment (PR: 1.74; 95% CI: 1.30–2.33); reporting barriers to accessing healthcare (PR: 1.55; 95% CI: 1.25–1.93); HIV seropositivity (PR: 1.35; 95% CI: 1.08–1.67); a history of non-fatal overdose (PR: 1.60; 95% CI: 1.32–1.95); and study enrolment in 2011 (PR: 1.39; 95% CI: 1.13–1.71).

**Table 1:** **Bivariate analyses of factors associated with reports of police beatings among IDU in Bangkok, Thailand (*n*** **= 639)**

**Characteristic**	**Ever beaten by police**		**Prevalence ratio (95% CI)**	***p - *value**
	**Yes**	**No**		
	**240 (37.6%)**	**399 (62.4%)**		
**Calendar year of study enrolment**				
2011	144 (43.4%)	188 (56.6%)	1.39 (1.13–1.71)	0.002
2009	96 (31.3%)	211 (68.7%)		
***Sociodemographic characteristics***				
**Age**				
< 37 years old	119 (38.3%)	192 (61.7%)	1.04 (0.85–1.27)	0.720
≥ 37 years old	121 (36.9%)	207 (63.1%)		
**Gender**				
Male	227 (46.7%)	259 (53.3%)	5.50 (3.24–9.33)	<0.001
Female	13 (8.5%)	140 (91.5%)		
Income from drug dealing*				
Yes	21 (43.8%)	27 (56.3%)	1.18 (0.84–1.65)	0.335
No	219 (37.1%)	372 (62.9%)		
***Drug use behaviour***				
**Heroin injection ever**				
Yes	228 (40.3%)	338 (59.7%)	2.45 (1.45–4.15)	<0.001
No	12 (16.4%)	61 (83.6%)		
**Midazolam injection ever**				
Yes	210 (40.0%)	315 (60.0%)	1.52 (1.10–2.10)	0.011
No	30 (26.3%)	84 (73.7%)		
**Methamphetamine injection ever**				
Yes	180 (39.6%)	274 (60.4%)	1.22 (0.96–1.55)	0.097
No	60 (32.4%)	125 (67.6%)		
**Crystal methamphetamine injection ever**				
Yes	48 (51.6%)	45 (48.4%)	1.47 (1.17–1.84)	<0.001
No	192 (35.2%)	354 (64.8%)		
**Syringe sharing ever**				
Yes	175 (47.0%)	197 (53.0%)	1.93 (1.52–2.45)	<0.001
No	65 (24.3%)	202 (75.7%)		
***Experiences with criminal justice system***				
**Ever in prison**				
Yes	220 (43.8%)	282 (56.2%)	3.00 (1.98–4.56)	<0.001
No	20 (14.6%)	117 (85.4%)		
**Ever in compulsory drug detention**				
Yes	56 (47.1%)	63 (52.9%)	1.33 (1.06–1.66)	0.012
No	184 (35.4%)	336 (64.6%)		
***Healthcare access***				
**Ever accessed methadone treatment**				
Yes	200 (42.2%)	274 (57.8%)	1.74 (1.30–2.33)	<0.001
No	40 (24.2%)	125 (75.8%)		
**Reporting barriers to accessing healthcare**				
Any	158 (44.6%)	196 (55.4%)	1.55 (1.25–1.93)	<0.001
None	82 (28.8%)	203 (71.2%)		
***Health outcomes***				
**HIV serostatus**				
Positive	61 (47.3%)	68 (52.7%)	1.35 (1.08–1.67)	0.007
Negative or unknown	179 (35.1%)	331 (64.9%)		
**Non-fatal overdose ever**				
Yes	81 (52.6%)	73 (47.4%)	1.60 (1.32–1.95)	<0.001
No	159 (32.8%)	326 (67.2%)		

*IDU* people who inject drugs, *CI* Confidence Interval.

* denotes events/activities in the previous 6 months.

Table 2 shows the results from the final multivariate log-binomial regression model. As shown, an interaction was found with median age and a history of methamphetamine injection. Reports of police beatings were independently and positively associated with younger age (< 37 years) among those who never injected methamphetamine (adjusted prevalence ratio [APR]: 1.69; 95% CI: 1.17–2.43); male gender (APR: 4.43; 95% CI: 2.63–7.49); a history of syringe sharing (APR: 1.44; 95% CI: 1.15–1.80); a history of incarceration (APR: 2.51; 95% CI: 1.68–3.77); a history of compulsory drug detention (APR: 1.22; 95% CI: 1.05–1.40); reporting barriers to accessing healthcare (APR: 1.23; 95% CI: 1.01–1.49); and study enrolment in 2011 (APR: 1.27; 95% CI: 1.07–1.49).

**Table 2:** **Multivariate log-binomial regression analysis of factors associated with reports of police beatings among IDU in Bangkok, Thailand (*n*** **= 639)**

**Variable**	**Adjusted PR**	**95% CI**	***p - *value**
**Calendar year of study enrolment**			
(2011 *vs.* 2009)	1.27	(1.07–1.49)	0.005
**Younger age among those who ever injected methamphetamine**			
(< 37 years *vs.* ≥ 37 years old)	1.18	(0.99–1.41)	0.062
**Younger age among those who never injected methamphetamine**			
(< 37 years *vs.* ≥ 37 years old)	1.69	(1.17–2.43)	0.005
**Gender**			
(Male *vs.* Female)	4.43	(2.63–7.49)	<0.001
**Syringe sharing ever**			
(Yes *vs*. No)	1.44	(1.15–1.80)	0.002
**Ever in prison**			
(Yes *vs*. No)	2.51	(1.68–3.77)	<0.001
**Ever in compulsory drug detention**			
(Yes *vs*. No)	1.22	(1.05–1.40)	0.008
**Reporting barriers to accessing healthcare**			
(Any *vs*. None)	1.23	(1.01–1.49)	0.043
**Non-fatal overdose ever**			
(Yes *vs*. No)	1.14	(0.97–1.34)	0.115

*IDU* people who inject drugs, *PR* prevalence ratio, *CI* Confidence Interval.

In sub-analysis, among participants completing surveys in 2011 (*n* = 144), 68.1% reported experiencing police beating while being interrogated, 43.1% reported being beaten during their arrest, 22.9% were beaten while being searched, and 22.9% reported having been beaten while in police holding cells.

## Discussion

We found that over one-third of a sample of IDU in Bangkok reported having ever been beaten by police. Reports of police beating were independently associated with study enrolment in 2011, male gender, younger age among those who never injected methamphetamine, a history of incarceration, compulsory drug detention and syringe sharing, and reporting barriers to accessing healthcare. Participants most commonly experienced police beating during the interrogation process.

To our knowledge, the present study is the first to quantitatively examine the prevalence and correlates of experiencing physical violence at the hands of police among IDU in Thailand. The findings that the overall prevalence was as high as 37.6% in 2009–2011, and the majority of the victims (68.1%) experienced it during the interrogation process raise serious concern about widespread police-perpetrated abuses against this population. Our findings are consistent with previous reports during the 2003 “war on drugs” campaign indicating that police beating was used as a tactic to extract confessions of drug-related crimes from suspected drug users [13]. We also found persistent reports of recent experiences with police beatings during the two-year study period. Furthermore, in a multivariate analysis, after extensive adjustment for social, demographic and behavioural factors, study enrolment in 2011 remained independently associated with reports of police beatings. Although the present study did not set out to assess the incidence of police beatings, these findings suggest that this form of police violence has continued in recent years.

We also found that male IDU experienced police beating more often than women. Thai police are believed to profile IDU based on factors such as track marks [13]. As the great majority of Thai IDU population is believed to be comprised of males [29], male IDU may be more susceptible to police profiling of IDU and police-perpetrated physical violence. However, it is also important to note that women may have been susceptible to other forms of police violence, such as sexual violence, which were not examined in the present study. We also found that younger IDU who never injected methamphetamine were more likely to have been beaten by police. Given that young people are a major target of drug demand reduction efforts in Thailand [30], young IDU may be more vulnerable to police beatings. However, the reasons why this association was found only among those who never injected methamphetamine remain unknown. Future research should seek to explore the context of police beatings in more depth.

Of particular concern is the finding that reports of police beating were independently associated with a history of incarceration and compulsory drug detention. This finding, considered alongside our data concerning the circumstances of police beatings, suggests that IDU in this setting may typically experience police beating before being sent to prison or compulsory drug detention. This is concerning because these institutions may be ill equipped to deal with physical and psychological manifestations of traumatic injuries [8]. Alternatively, the finding may suggest that individuals with a history of incarceration or compulsory drug detention are easier targets for police. Indeed, previous reports documented widespread use of “blacklists” by the Thai police during the 2003 “war on drugs” campaign, on which individuals with records of drug-related arrests were listed as suspected drug users or traffickers [13]. More recent reports also suggested the continued use of blacklists by police [18,31], indicating that the latter interpretation may be also plausible.

Importantly, episodes of police beating were also independently associated with syringe sharing and reporting barriers to accessing health services. Consistent with previous studies from other settings indicating the negative impact of aggressive drug law enforcement on seeking health and harm reduction services by IDU [2,19,20], our findings may suggest that individuals who experienced police beating may have retreated into more hidden settings where it was difficult to obtain sterile syringes, making the adoption of harm reduction practices become difficult or impossible. Several lines of evidence support such negative impacts of police beatings on HIV risk behaviour among IDU. Specifically, previous studies from Ukraine also found a significant association between police beatings and syringe sharing among IDU [32,33], and mathematical modelling showed that the elimination of police beatings could substantially avert HIV infection in this population [33]. In addition, a recent study of IDU in Bangkok demonstrated that past experiences with police beatings were independently associated with recent syringe sharing, indicating a lasting effect of police beatings on HIV risk behaviour among IDU in this setting [34]. It may also be that acts of violence by police create environment that promotes fear and constrains Thai IDUs’ access to healthcare. However, as the present study did not assess the temporal relationship between police beatings and health-seeking practices, future research should further examine this relationship in this setting.

Our findings have implications for policies and programs related to drug law enforcement in Thailand. First, the extent of police beating reported in the present study raise concern about widespread violations of basic human rights of IDU in this setting, including the rights to security of the person (Article 9) and to freedom from torture and cruel, inhuman, and degrading treatment (Article 7) under the International Covenant on Civil and Political Rights, to which Thailand became a party in 1996. The use of torture is also prohibited under Section 32 of the *Constitution of the Kingdom of Thailand B.E. 2550*[35]. In keeping with the law, a greater oversight of police operations should be a priority for the Thai government. Second, given the findings that police beating may be undermining HIV prevention among IDU in this setting, more efforts should be made to harmonize drug law enforcement activities and public health goals. Some examples of such efforts proposed and made in other settings include encouraging police officers to exercise discretion or cautioning or other measures instead of arresting street-level drug users [36]; training police officers to engage in or at least not undermine harm reduction activities [37,38]; and establishing multi-sectoral partnerships between police and health agencies [38,39]. However, evaluations of these efforts showed some mixed results, pointing out various barriers to implementation, including personnel transfers, police culture, variations in public perception of the role of police, and ongoing police corruption [2,39,40]. There is clearly a need for more work in this area. Lastly, ensuring access to legal services among IDU is important in order to help victims of police abuse obtain redress and compensation in this setting. In addition, a recent review indicated that expanded legal services also have potential to prevent police abuse and promote health benefits among IDU [41].

This study has several limitations. First, due to the cross-sectional study design, we were unable to assess temporal relationships between the outcome and explanatory variables. Second, the self-reported data may have been affected by socially desirable responding or recall bias. Third, as the study sample was not randomly selected, our findings may not be generalizable to other populations of IDU in Thailand.

## Conclusions

In sum, we found that a high proportion of a community-recruited sample of IDU in Bangkok reported having been beaten by police. Experiencing police beating was independently associated with indicators of drug-related harm, including syringe sharing and barriers to accessing healthcare. These findings suggest that the over-reliance on law enforcement-based approaches may be contributing to human rights violations at the hands of police and exacerbating HIV risk among IDU in this setting. Therefore, they indicate the need for greater police oversight and a shift toward the implementation of evidence-based policies and programs specific to HIV/AIDS and illicit drug use.

## Competing interests

The authors declare that they have no competing interests.

## Authors’ contributions

KH and TK designed the study. KH conducted the statistical analyses, drafted the manuscript, and incorporated suggestions from all co-authors. All authors made significant contributions to the conception of the analyses, interpretation of the data, and drafting of the manuscript. All authors read and approved the final manuscript.

## Pre-publication history

The pre-publication history for this paper can be accessed here:

http://www.biomedcentral.com/1471-2458/13/733/prepub
